# Interaction of Bisphenol A and Its Analogs with Estrogen and Androgen Receptor from Atlantic Cod (*Gadus morhua*)

**DOI:** 10.1021/acs.est.4c01500

**Published:** 2024-08-01

**Authors:** Siri Øfsthus Gokso̷yr, Fekadu Yadetie, Christine Tveiten Johansen, Rhîan Gaenor Jacobsen, Roger Lille-Lango̷y, Anders Gokso̷yr, Odd André Karlsen

**Affiliations:** Department of Biological Sciences, University of Bergen, Bergen N-5020, Norway

**Keywords:** reporter gene assays, *in vitro*, precision-cut liver slices, *ex vivo*, vitellogenin

## Abstract

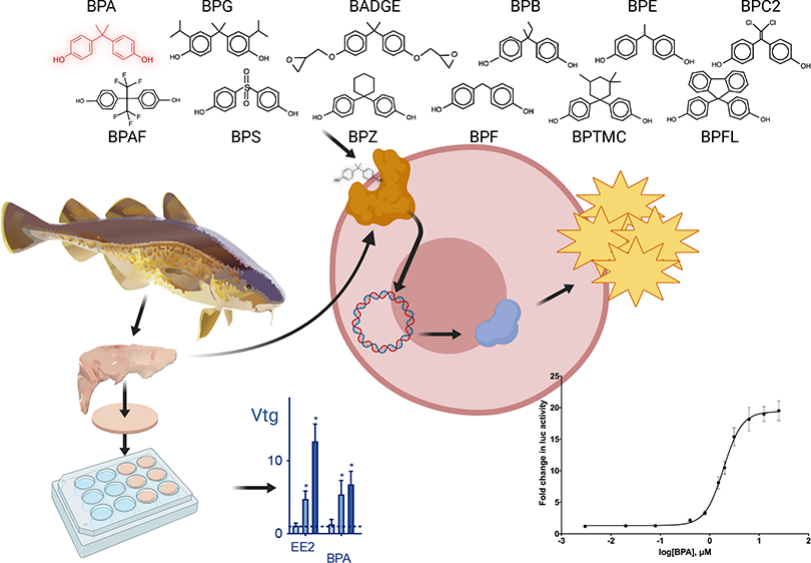

The widespread use of bisphenol A (BPA) in polycarbonate plastics and epoxy resins has made it a prevalent environmental pollutant in aquatic ecosystems. BPA poses a significant threat to marine and freshwater wildlife due to its documented endocrine-disrupting effects on various species. Manufacturers are increasingly turning to other bisphenol compounds as supposedly safer alternatives. In this study, we employed *in vitro* reporter gene assays and *ex vivo* precision-cut liver slices from Atlantic cod (*Gadus morhua*) to investigate whether BPA and 11 BPA analogs exhibit estrogenic, antiestrogenic, androgenic, or antiandrogenic effects by influencing estrogen or androgen receptor signaling pathways. Most bisphenols, including BPA, displayed estrogenic properties by activating the Atlantic cod estrogen receptor alpha (gmEra). BPB, BPE, and BPF exhibited efficacy similar to or higher than that of BPA, with BPB and BPAF being more potent agonists. Additionally, some bisphenols, like BPG, induced estrogenic effects in *ex vivo* liver slices despite not activating gmEra *in vitro*, suggesting structural modifications by hepatic biotransformation enzymes. While only BPC2 and BPAF activated the Atlantic cod androgen receptor alpha (gmAra), several bisphenols exhibited antiandrogenic effects by inhibiting gmAra activity. This study underscores the endocrine-disrupting impact of bisphenols on aquatic organisms, emphasizing that substitutes for BPA may pose equal or greater risks to both the environment and human health.

## Abstract 2

This study substantiates that bisphenols replacing BPA can exhibit similar or greater endocrine disruptive properties and represent the first comprehensive study analyzing the effects of BPs on both the androgen and estrogen receptor in fish.

## Introduction

Bisphenol A (BPA) is a high-production volume chemical that has been used as a synthetic additive in polycarbonate plastics and epoxy resins since the 1940s. Today, BPA is commonly found in consumer products such as food and beverage containers, baby bottles, lining of metal cans, water pipes, dental monomers, and thermal paper, among many others.^1,2^ BPA is also released in large quantities to freshwater and marine systems from effluent discharges in manufacturing plants, wastewater treatment plants, landfill sites, and from leaching of BPA-based products.^3−5^ Even though BPA has a relatively short lifetime in nature,^6^ the persistence of plastic waste and the continuous effluent discharges to the environment means that aquatic species are consistently exposed to BPA.^7,8^ Accordingly, a recent study demonstrated that BPA can leach from marine microplastics and accumulate in wild fish.^9^

The major concern regarding BPA is attributed to its endocrine-disrupting properties mediated through binding to steroid hormone receptors, such as the estrogen (ER) and androgen (AR) receptors.^10−15^ By modulating the activities of ER and AR, BPA can affect the endocrine regulation of the hypothalamic-pituitary–gonadal axis (HPG-axis) and impair reproductive functions in organisms. In fish, BPA has been shown to affect gonadal maturation, reduce androgen levels, and alter spermatogenesis.^16−19^ Recent reports have also demonstrated that BPA can bind to other nuclear receptors, including estrogen-related receptor gamma (ERRG), pregnane X receptor (PXR), thyroid hormone receptor (TR), and the peroxisome proliferator-activated receptor gamma (PPARG), and has thus a potential of affecting numerous signaling pathways and physiological processes.^12,20−22^

Due to the growing concern of its endocrine-disrupting properties, BPA has become more strictly regulated, with EFSA recently lowering the tolerable daily intake of BPA from 4 μg to 0.2 ng per kg body weight.^23^ However, the restrictions on BPA usage have led manufacturers to develop alternative bisphenols (BPA analogs) that are presently largely unregulated. BPA analogs are chemicals harboring the same physicochemical properties and similar structures as BPA. These compounds have among others been detected in personal care products, paper products, foodstuffs, and indoor dust.^24,25^ Analyses of sediment, water, and aquatic organisms show that BPA analogs also are present in the environment.^26−28^ Some of these compounds are more resistant to biodegradation than BPA, making them more susceptible to accumulate in aquatic environments and aquatic species.^29−31^ Importantly, recent studies indicate that many of the BPA analogs have endocrine disrupting potential similar to those of BPA.^18,32^

Atlantic cod (*Gadus morhua*) is an ecologically and commercially important teleost species in the North Atlantic Ocean. It has commonly been used in environmental monitoring programs of marine ecosystems, including the OSPAR convention and water column monitoring of offshore petroleum activities in Norwegian waters.^33−35^ The genome sequence of Atlantic cod was published in 2011,^36^ facilitating the emergence of this species as a model in toxicological studies of cold-water marine teleosts, including genome-wide transcriptomics and proteomics studies as well as the development of *in vitro* and *ex vivo* assays for studying effects of environmental pollutants at the molecular and tissue level.^33,37−43^ The population of the Norwegian coastal cod has declined dramatically over the last decades.^44,45^ Although the reason for this decline is most likely complex and multifaceted, exposure to both chemicals of emerging concern (CEC) and legacy pollutants may be contributing factors that affect the survival, growth, and reproduction success of teleosts.^46−48^ Importantly, recent environmental monitoring programs of marine species from the inner Oslo Fjord (Norway) have reported the presence of BPA and BPA analogs in cod liver and bile, including bisphenols that are currently replacing BPA such as BPF and BPS. These findings underscore an ongoing exposure of Atlantic cod and marine organisms residing in these areas to potentially potent endocrine disruptors.^49,50^ In general, the available data on levels of bisphenols in fish is sparse. Staniszewska et al. found significant levels of BPA in the liver and muscle of herring, flounder, and Atlantic cod from the Southern Baltic Sea,^51^ and Akhbarizadeh et al. analyzed fish from the Persian Gulf, finding highest levels of BPA and BPB, especially in fish species at the higher trophic levels.^52^ Both of these studies indicated the biomagnification of bisphenols in the food chain.

The objective of this study was to deepen our mechanistic understanding of how bisphenol A (BPA) and 11 BPA analogs can impact the estrogenic and androgenic signaling pathways in ecologically important fish species. To achieve this, *in vitro* reporter gene assays using Atlantic cod estrogen receptor alpha (Era) and androgen receptor alpha (Ara), as well as vitellogenin (Vtg) assays in *ex vivo* precision-cut liver slice (PCLS) cultures, were performed. Results indicate that the majority of bisphenols were able to modulate the activities of these hormone receptors *in vitro* and activate the Vtg synthesis in PCLS cultures. Notably, the most active compounds in the *in vitro* reporter gene assays primarily exhibited Era activation, while several bisphenols produced antagonistic effects on Ara.

## Materials and Methods

### Chemicals

17α-Ethynylestradiol (EE2, CAS no. 57–63–6, purity >98%), testosterone (CAS no. 58–22–0, purity >98%), tamoxifen (CAS no. 10540–29–1, purity >99%), flutamide (CAS no. 13311–84–7, purity >99%), BPA (CAS no. 80–05–7, purity >99%), BPB (CAS no. 77–40–7, purity >98%), BPC2 (CAS no. 14868–03–2, purity >98%), BPE (CAS no. 2081–08–5, purity >98%), BPF (CAS no. 620–92–8, purity >98%), BPG (CAS no. 127–54–8, purity >98%), BPS (CAS no. 80–09–1, purity >98%), BPZ (CAS no. 843–55–0, purity >99%), BPAF (CAS no. 1478–61–1, purity 97%), BPFL (CAS no. 3236–71–3, purity >99%), BPTMC (CAS no. 129188–99–4, purity >97%), and BADGE (CAS no. 1675–54–3) were purchased from Sigma-Aldrich (St. Louis, MO, USA). Methanol was used to dissolve BPB, while the other chemicals were dissolved in dimethyl sulfoxide (DMSO).

### Atlantic Cod

Atlantic cod (*Gadus morhua*) were obtained from Havbruksstasjonen in Tromso̷ AS (Nofima, Tromso̷, Norway) and kept in 500 L tanks with running seawater at 10 °C and 12 h light/12 h dark cycle at the Industrial and Aquatic Laboratory (Bergen, Norway). The fish were fed a commercial diet (Amber Neptune, batch no. 3343368, Skretting, Stavanger, Norway). Male fish of approximately 1.5 to 2 years old and bw of 1099 ± 315g (mean ± standard deviation) were used for the *ex vivo* experiments. The experiment was performed in accordance with the guidelines and with the approval of the Norwegian Board of Biological Experiments with Living Animals (FOTS ID 11730).

### Cloning of Estrogen Receptor 1 (esr1) and Androgen Receptor (ara) cDNA

Total RNA was extracted from Atlantic cod liver and testis tissue using the RNeasy Plus Universal Mini Kit (Qiagen). cDNA was synthesized using the iScript synthesis kit (BioRad) and Superscript III (Invitrogen) with RNA isolated from the liver and testis, respectively. Nucleotide sequences encoding the hinge region and the ligand binding domain (LBD) of Atlantic cod Era (gmEra, JX178935, aa 170–455) and Ara (gmAra, FJ268742, aa 409–718) were amplified by PCR (Phusion High-Fidelity DNA Polymerase (M0530)) from cDNA prepared from liver and testis, respectively, and subcloned into the pSC-A (Strataclone) plasmid. *Bam*HI and *Eco*RI sites were introduced in the primers used in the PCR (Table S1) for transferring the encoding fragments from pSC-A into the pCMX-GAL4-DBD eukaryotic expression plasmid.^53^

### Cell Culturing, Luciferase Reporter Gene Assay, and Cell Viability

COS-7 cells were maintained in a humidified incubator with 5% carbon dioxide (CO_2_) at 37 °C in phenol red Dulbecco’s modified Eagle’s medium (DMEM) (Sigma-Aldrich), supplemented with 10% fetal bovine serum (FBS), 1 mM sodium pyruvate (Thermo Scientific), 4 mM l-glutamine (Sigma-Aldrich), and penicillin-streptomycin. Cells were subcultured when confluency reached 80–90% by dissociating cells with trypsin-EDTA.

Ligand activation of gmEra and gmAra was studied with a luciferase reporter gene assay in COS-7 cells transiently transfected with the pCMX-GAL4(DBD)-gmEra(LBD) or pCMX-GAL4(DBD)-gmAra(LBD) plasmid by using the TransIT-LT1 reagent (TransIT-LT1 Transfection Reagent, Mirus). Cells were seeded into 96-well plates (5000 cells/well) and incubated for 24 h before cotransfection with the plasmid mixture (100 ng/well). The plasmid mixture contained GAL4-UAS luciferase reporter plasmid (tk-(MH100)x4 luc), the pCMX-GAL4(DBD)-gmEra(LBD) or pCMX-GAL4(DBD)-gmAra(LBD) receptor plasmids (2:1 reporter:receptor plasmid mass ratio), and a plasmid constitutively producing the β-galactosidase enzyme used for normalization of transfection efficiency (pCMV- β-Gal). Twenty-four h post-transfection, the cells were exposed to test compounds dissolved in DMSO or methanol (for BPB) for 24 h. The maximum DMSO/methanol concentrations in the exposure wells were 0.5% (v/v) in DMEM medium without phenol red (Sigma-Aldrich). After exposure, cells were lysed, and luciferase and beta-galactosidase activities were measured as luminescence and absorbance (420 nm) after the addition of luciferin and ONPG substrates, respectively, using an Enspire Multimode plate reader (PerkinElmer, Waltham, Massachusetts, USA).

The cytotoxicity of the test compounds toward COS-7 cells was monitored by measuring the metabolic activity and membrane integrity through the conversion of resazurin and 5-carboxyfluorescein diacetate acetoxymethyl ester (CFDA-AM), respectively, as described previously by Pérez-Albaladejo et al.^54^

### SDS-PAGE and Western Blotting

SDS-PAGE (sodium dodecylsulfate polyacrylamide gel electrophoresis) and western blot analysis were performed essentially as described by Towbin et al.^55^ COS-7 cells were cultivated in 6-well plates (6 × 10^5^ cells/well) and transiently transfected with either gmEra (pCMX-GAL4(DBD)-gmEra(LBD)) or gmAra (pCMX-GAL4(DBD)-gmAra(LBD)) plasmids for 24h, as well as including untransfected control cells. The transfection protocol was similar to that used in the luciferase reporter gene assay but with a plasmid mix concentration relating to the higher volume and increased cell number in the wells (plasmid mix corresponding to a total of 2500 ng DNA/well). The cells were harvested by scraping, lysed in SDS-PAGE sample buffer, and heated at 90 °C for 10 min for denaturation of proteins. Approx. 10 μg of protein was loaded into each well of the SDS-PAGE gel. After electroblotting, the nitrocellulose membrane was blocked with 5% dry milk in TBS-Tween, and a mouse anti-Gal4-DBD primary antibody (Santa Cruz Biotechnology) was added followed by a secondary HRP-linked sheep-antimouse IgG antibody (GE Healthcare). Immunoreactive proteins were visualized with Enhanced Chemiluminescence (GE Healthcare) and a Gel-Doc (Bio-Rad) instrument. Anti-actin antibody (Abcam) was used as a loading control.

### Culturing of Precision-Cut Liver Slices (PCLS) and Exposure Assays

PCLS culturing and exposure assays were performed essentially as described previously,^42,56^ with the modifications described below. Juvenile Atlantic cod were killed by a blow to the head, and the liver was dissected out and placed in a Petri dish containing ice-cold PCLS buffer (122 mM NaCl, 4.8 mM KCl, 1.2 mM MgSO_4_, 11 mM Na_2_HPO_4_, 3.7 mM NaHCO_3_, pH 8.4). The liver was then cut into small tissue blocks (approximately 3 cm long, 2 cm wide, and 1–3 cm high) and kept in ice-cold complete culture medium (L-15 medium supplemented with 10% FBS and 1× antibiotic-antimycotic solution). For slicing, the tissue block was fixed on the specimen plate of a Leica vibrating blade microtome VT1200 (Leica, Wetzlar, Germany) using Loctite Super Glue (Clas Ohlson, Bergen, Norway), and 250 μm thick tissue slices were cut (under ice-cold PCLS buffer) and kept in a Petri dish with ice-cold complete culture medium. Using a sterile razor blade, the tissue slices (2 × 3 cm) were then divided into smaller pieces (approximately 4 × 4 mm) and distributed into a 12-well cell culture plate (Sarstedt, Nümbrecht, Germany), each well containing 6 slices and 2 mL of complete culture medium. The PCLS were preincubated at 10 °C for 2 h before exposure. After preincubation, 1 mL of the medium was removed from each well and replaced by 1 mL of fresh complete culture medium containing 2x the final desired concentration and incubated at 10 °C for 96 h with shaking at 50 rpm. All wells, including the vehicle control, contain equal concentrations of DMSO (methanol for BPB), not exceeding 0.5% (v/v). At the end of the exposure, slices were collected with tweezers and rinsed briefly in cold PBS. Excess PBS was removed by carefully touching a dry surface of the culture plate with the slice 4 times before measuring the weight in a preweighed cryotube. The slices were then snap-frozen in liquid N_2_ and stored at −80 °C. The media was also sampled and stored at −80 °C for viability (LDH) assay and vitellogenin ELISA.

### Vitellogenin Enzyme-Linked Immunosorbent Assay (ELISA)

Vitellogenin (Vtg) in media collected from exposed PCLS was quantified using the Atlantic Cod Vitellogenin ELISA kit (Biosense Laboratories AS, Bergen, Norway) according to manufacturer’s instructions. Vtg was quantified in 100 μL of undiluted media and normalized to the weight of the liver tissue. Statistical analysis of ELISA data was performed with GraphPad Prism Software version 9 (GraphPad Prism, La Jolla, CA, USA) by using one-way ANOVA followed by Dunnett’s test (for normally distributed data) or Friedman test followed by Dunn’s test (for non-normally distributed data), as described previously for PCLS experiments (Yadetie et al.^42^).

### Monitoring of PCLS Viability with the Lactate Dehydrogenase (LDH) Assay

To monitor the viability of the liver tissue, the release of LDH to the medium was quantified with an LDH Cytotoxicity Detection kit (Roche, Basel, Switzerland) according to the manufacturer’s protocol. Briefly, 50 μL of PCLS medium collected at the end of 96 h exposure was pipetted in triplicates into a clear 96-well plate, 50 μL of provided reagent mixture with the substrate was added, and the mixture was incubated for 10 min at room temperature in the dark. Absorbance was measured at 490 nm using an EnSpire plate reader (PerkinElmer, Waltham, USA), with 650 nm reference measurement. The LDH activity was normalized to the liver tissue weight, and statistical analysis was performed as described above for the ELISA data.

## Results

### Estrogenic and Antiestrogenic Activity of BPs

**Figure 1:**
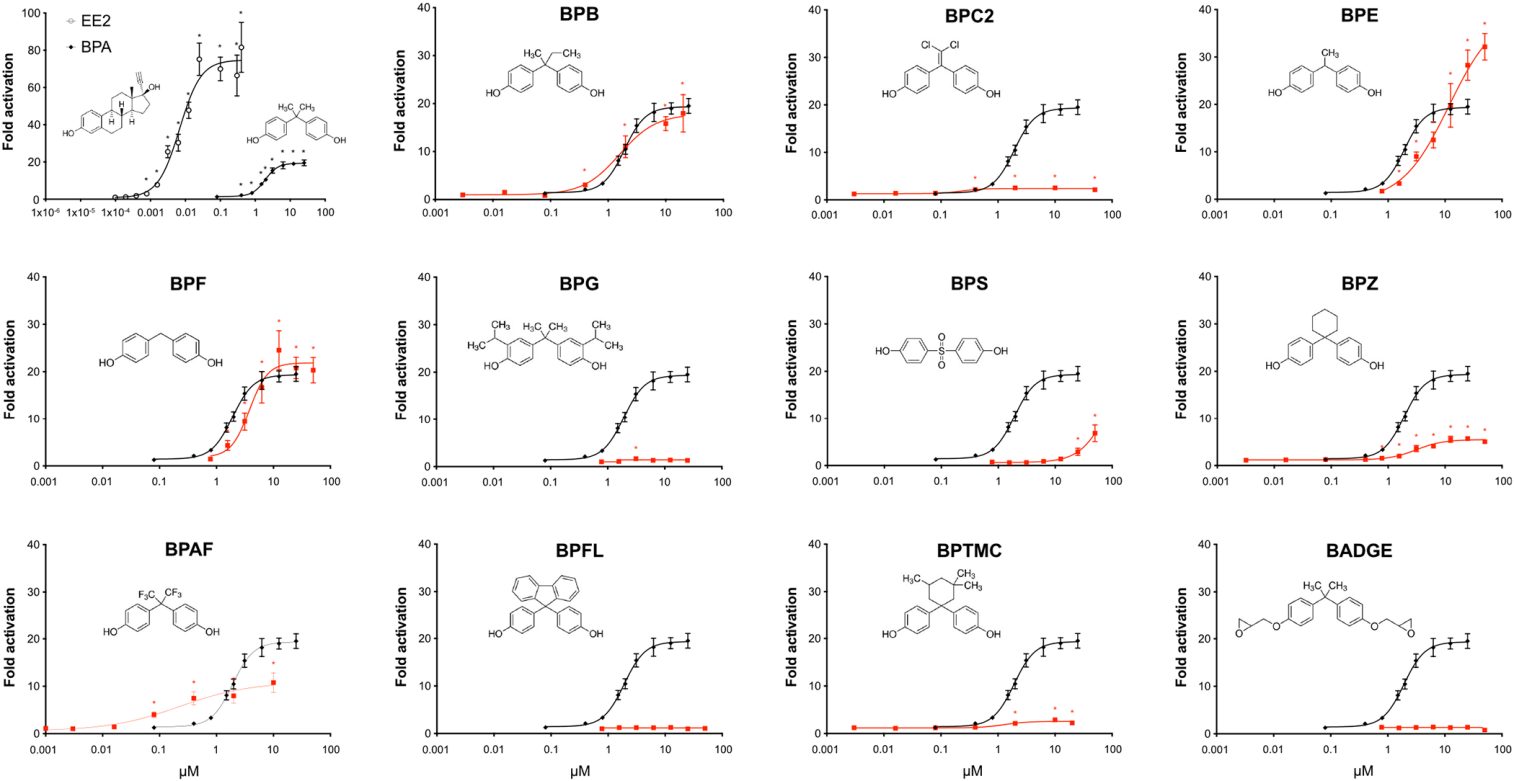
Estrogenic activity of bisphenols. COS-7 cells transiently transfected with gmEra-LBD were exposed to EE2 (open circles), BPA (black circles), and 11 different BPA analogs (red squares and as indicated) at increasing concentrations. Responses are presented as mean fold activation in luciferase activity in comparison to solvent-exposed cells ± SEM from three independent experiments with three technical replicates (*n* = 9). The dose–response curves were created using nonlinear regression and the four parameter logistic fit in Graphpad Prism v.9. For comparison, the dose–response curve of BPA (black line) is indicated together with each individual BP tested (red line). *p < 0.05 versus solvent control by one-way ANOVA and Dunnett’s multiple comparison post hoc test.

Luciferase reporter gene assays were established with COS-7 cells transiently producing the LBD of gmEra (Figure S1) for evaluating the estrogenic potential of 12 bisphenols. EE2 was initially used as a well-known Era agonist for the assay, producing a maximum response (*E*_max_) of 80-fold activation compared to the solvent control and an EC_50_-value of 6.8 nM (Figure 1). BPA also transactivated gmEra but exhibited both a lower efficacy (19.4-fold activation) and a higher EC_50_-value (1.9 μM) in comparison to EE2, which was close to 10^3^ more potent than BPA (Figure 1). Importantly, among the BPA analogs tested, eight compounds, including BPB, BPC2, BPE, BPF, BPS, BPZ, BPAF, and BPTMC, activated gmEra more than 1.5-fold (Figure 1 and Table 1). Both BPB and BPF produced very similar dose–response curves as BPA, while BPE demonstrated a higher maximum fold activation, although it was less potent than BPA. BPAF was the most potent bisphenol compound but produced a lower efficacy in comparison to BPA. Notably, BPG, BPFL, and BADGE did not activate gmEra in this assay. Based on the transactivation profiles, the following ranking of the BPA analogs was made according to their *E*_max_ values BPE > BPF > BPA > BPB > BPAF > BPS > BPZ > BPTMC > BPC2. Their potencies toward gmEra (EC_50_-values) were ranked as follows: BPAF > BPC2 > BPTMC > BPB > BPA > BPZ > BPF > BPE. See Table 1 for a summary of the potencies, efficacies, and relative potencies (REP) of the compounds tested.

**Table 1:** Summary of Potencies and Efficacies of BPA and Its Analogs for gmEra and gmAra as Determined with Luciferase Reporter Gene Assays^b^

	gmEra					gmAra			
	*E*_max_	EC_50_ (μM)	REP_50_	IC_50_ (μM)		*E*_max_	EC_50_ (μM)	REP_50_	IC_50_ (μM)
EE2	74	0.007	271	na		na	na	na	na
T	na	na	na	na		42.1	0.01	3210	na
BPA	19.4*	1.9*	1.0			1.6^a^	25.7^a^	1.0	0.14*
BPB	17.8*	1.6*	1.2	∼0.10		1.2			0.14*
BPC2	2.4*	0.2	9.5	0.06*		2.3^*^^a^	∼4343^a^	0.01	0.10*
BPE	32.2^*^^a^	11.7^*^^a^	0.2			1.2^a^			0.46*
BPF	21.9*	3.8*	0.5			1.4			1.07*
BPG	∼1.4			1.40*		0.8			0.20*
BPS	6.8^*^^a^	83.3^*^^a^	0.02			1.4			
BPZ	5.5*	3.13*	0.6	3.03*		0.8			0.88*
BPAF	10.9*	0.23*	8.3			2.2*	25.0*	1.0	0.03*
BPFL						1.3			
BPTMC	2.56*	1.28	1.5	∼1.86*		1.4			1.75*
BADGE	1.3*					1.0			
tamoxifen	na	na	na	0.002*		na	na	na	na
flutamide	na	na	na	na		na	na	na	∼0.71*

a A plateau of activation was not reached. *E*_max_ values are the highest fold activation obtained. EC_50_ values were calculated in Graphpad Prism (v.9) using nonlinear regression and the four-parameter logistic fit.

b Abbreviations: REP_50_, relative potency EC_50_ (EC_50_-bisphenol A/EC_50_-bisphenol X); EE2, ethynylestradiol; T, testosterone; na, not assessed. *, Statistically significant activation or inhibition.

**Figure 2:**
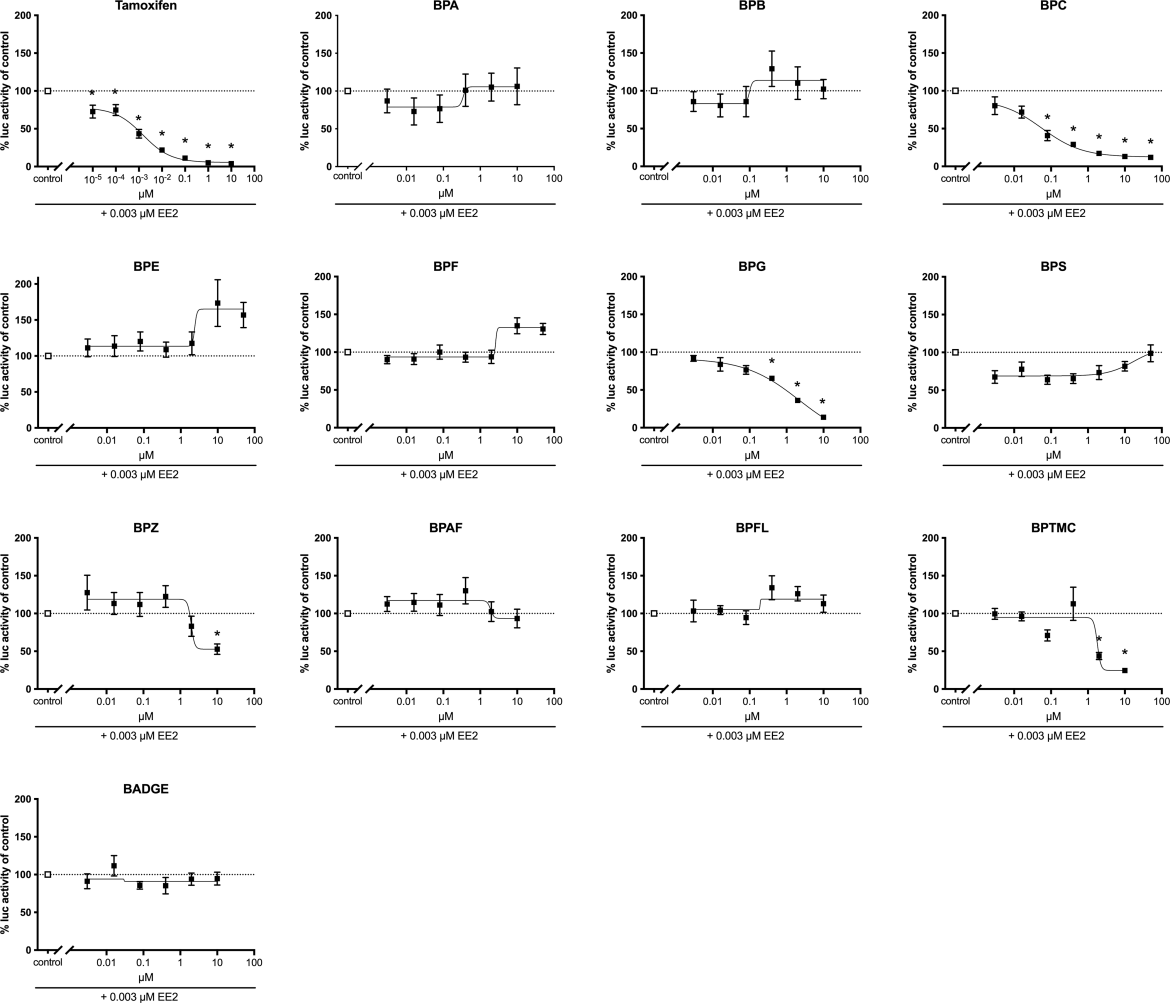
Antiestrogenic effects of bisphenols. COS-7 cells transiently producing gmEra LBD were coexposed with a binary mixture consisting of a fixed concentration of EE2 (3 nM) and increasing concentrations (0.003–50 μM) of the individual bisphenol analogs or tamoxifen (0.00001–10 μM), as indicated. Responses in coexposed cells are presented relative to responses in cells exposed solely to EE2 (adjusted to 100%) ± SEM from three independent experiments (two for tamoxifen) with three technical replicates. The dose–response curves were created using nonlinear regression and the four-parameter logistic fit in Graphpad Prism v.9. *p < 0.05 versus solvent control by ANOVA and Dunnett’s multiple comparison post hoc test.

To investigate potential antiestrogenic effects, COS-7 cells transiently expressing gmEra LBD were exposed to the 12 bisphenols in binary mixtures with a fixed concentration of EE2 (3 nM, corresponding to EC_20_) (Figure 2 and Table 1). Tamoxifen is a well-characterized antagonist for mammalian and fish ER/Er, and significant inhibition of EE2-mediated gmEra activation was observed at low concentrations (0.01 nM), with an IC_50_ determined to be 1.5 nM. As expected, BPA did not antagonize gmEra. However, we observed significant antagonizing effects of BPC2, BPG, BPZ, and BPTMC, with calculated IC_50_-values of 0.06, 2.2, 2.0, and 1.8 μM, respectively. The remaining BPs did not antagonize the EE2-mediated Era activity (Table 1). In fact, some of these Era agonists, such as BPE and BPF, show trends of additive effects with EE2 (Figure 2). To evaluate if the observed decrease in EE2-mediated Era activity was due to cytotoxicity, the cell viability of the COS-7 cells was monitored by recording the metabolic activity and membrane integrity using resazurin and CFDA-AM assays, respectively (Figure S2). These assays revealed that COS-7 cells exposed to a binary combination of 0.003 μM EE2 and the bisphenols BPG, BPZ, and BPTMC affected the cell viability at the highest concentrations used (50 μM) in the reporter gene assay, supporting that the observed decrease in EE2-mediated gmEra activities that occurred in a dose-dependent manner at lower concentrations is attributed to antagonistic properties of these BPs. Concentrations that reduced cell viability were accordingly not included in the preparation of the concentration–response curves presented in Figure 2.

### Androgenic and Antiandrogenic Activity of BPs

**Figure 3:**
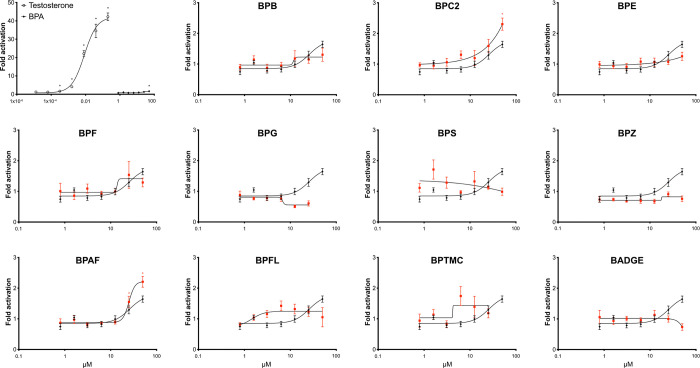
Androgenic activity of bisphenols. COS-7 cells were transiently transfected with gmAra LBD and exposed individually to 12 bisphenol analogs at increasing concentrations. Responses in exposed cells are presented as mean fold activation in luciferase activity in comparison to solvent control ± SEM from three independent experiments with three technical replicates (*n* = 9, *n* = 12 for testosterone). The dose–response curves were created using nonlinear regression and the four-parameter logistic fit in Graphpad Prism v.9. For comparison, the dose–response curve of BPA (black line) is indicated together with each individual BP tested (red line). *p < 0.05 versus solvent control by one-way ANOVA and Dunnett’s multiple comparison post hoc test.

Androgenic activities of the 12 BPs were assessed using the luciferase reporter gene assay with COS-7 cells transiently producing gmAra LBD (Figure 3, Table 1, and Figure S1). Testosterone (T) is an endogenous ligand of Ar and produced a maximal fold activation of 42.1, relative to the solvent control. The EC_50_ value was determined to be 8.5 nM. gmAra was significantly activated only by BPC2 and BPAF, demonstrating a similar maximal activation of 2.3-fold and 2.2-fold, respectively. However, we observed some trends in which the highest concentrations of many BPs slightly increased the gmAr activity. The EC_50_ value for BPAF was determined to be 25.0 μM. A summary of the data obtained with the different bisphenols toward gmAra, including REPs, is presented in Table 1.

**Figure 4:**
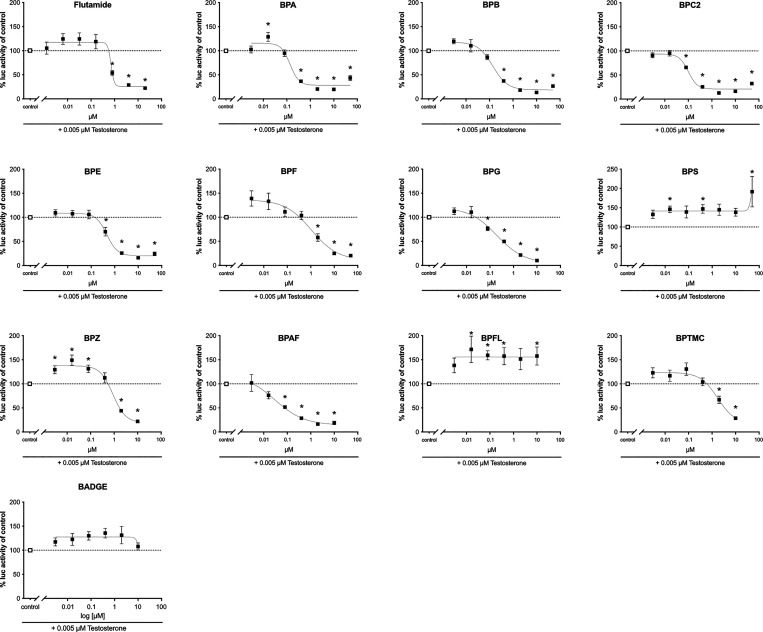
Antiandrogenic effects of bisphenols. COS-7 cells transiently transfected with gmAra LBD were exposed to a binary combination of a fixed concentration of 5 nM testosterone and increasing concentrations of bisphenols (0.003–50 μM) or flutamide (0.001–20 μM), as indicated. Responses in coexposed cells are presented relative to cells exposed to testosterone (adjusted to 100%) ± SEM from three independent experiments with three technical replicates (*n* = 9). The dose–response curves were created using nonlinear regression and the four-parameter logistic fit in Graphpad Prism v.9. *p < 0.05 versus solvent control by ANOVA and Dunnett’s multiple comparison post hoc test.

To evaluate the antiandrogenic activity of the BPs, COS-7 cells transiently transfected with gmAra LBD were exposed to the BP analogs (0.003–50 μM) in the presence of 5 nM testosterone (equivalent to EC_20_) (Figure 4 and Table 1). Flutamide was included as a known teleost Ar antagonist,^57^ producing an IC_50_ of 0.7 μM. Notably, BPA, BPB, BPC2, BPE, BPF, BPG, BPZ, BPAF, and BPTMC were all able to significantly decrease the testosterone-mediated Ara-activity (Figure 4). On the other hand, significant increases in gmAr activity were observed when coexposing cells with BPS and BPFL with testosterone, as well as for BPZ at the lower concentrations used (3–80 nM). A summary of the efficacies and potencies of the different compounds toward gmAra is given in Table 1. Cytotoxicity was monitored using the resazurin and CFDA-AM assays (see Figure S2), which revealed that BPG, BPZ, BPAF, BPTMC, and BADGE were cytotoxic at the highest concentration used (50 μM) in the reporter gene assay and therefore not included in the preparation of the concentration–response curves in Figure 4.

### Estrogenic Responses in Precision-Cut Cod Liver Slices (PCLS)

**Figure 5:**
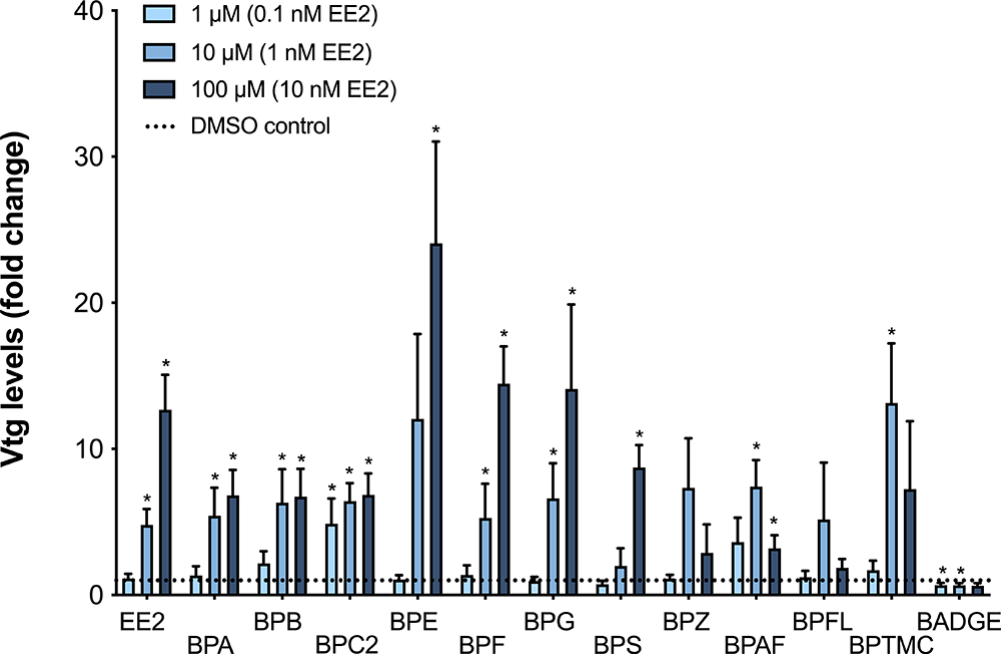
Relative vitellogenin production in Atlantic cod precision-cut liver slices (PCLS) exposed to bisphenols and EE2. PCLS were exposed to 1, 10, and 100 μM bisphenols or 0.1, 1, and 10 nM EE2, for 96 h. EE2 was included as positive control for activation of the gmEra signaling pathway. Levels of induced Vtg in the PCLS media were measured using ELISA (Cod Vitellogenin ELISA kit, Biosense Laboratories AS). The data represents mean ± SEM of fold change (compared to solvent control) of Vtg levels from 4 to 10 fish. *n* = 8–10 for all bisphenols except for BADGE (*n* = 4). Asterisks denote significant changes (*p* < 0.05) compared to solvent control.

To further explore the modulation of gmEra activity by BPA and its analogs, the bisphenol compounds were tested for their ability to activate the Era-signaling pathway *ex vivo* by using Atlantic cod PCLS. An ELISA assay was used to quantify the Er-mediated synthesis of Vtg in media obtained from exposed liver slices. As expected, EE2 exposure increased Vtg-levels in the media in a dose-dependent manner at nanomolar concentrations (Figure 5). Furthermore, nine of the 12 BPs were also able to increase the synthesis of Vtg *ex vivo*, although the increases observed with BPZ and BPFL were only moderate and not statistically significant in this assay (Figure 5). Notably, BPG, which did not activate the estrogen receptor *in vitro*, was able to induce Vtg synthesis in PCLS. Furthermore, BADGE did not induce increased levels of Vtg but instead produced a small, but significant, decrease in the Vtg synthesis. For many of the BPs activating Vtg synthesis, the responses tended to follow concentration–response relationships, except for BPZ, BPAF, BPFL, and BPTMC, where maximum Vtg levels were observed at the medium (10 μM) concentrations (Figure 5). The viability of the liver slices was monitored using a lactate-dehydrogenase (LDH) assay, and no significant increase in the LDH-activity was observed in these experiments (Figure S3).

## Discussion

BPA and 11 BPA analogs, including bisphenols that are currently replacing BPA as well as other analogs that potentially can be used as new alternatives in consumer and industrial products, were assessed for endocrine disrupting properties by modulating the transcriptional activities of Era and Ara in Atlantic cod. Estrogenic, antiestrogenic, androgenic, and antiandrogenic effects were assessed *in vitro,* while modulation of the estrogen signaling pathway was in addition analyzed *ex vivo* with PCLS.

### Agonistic and Antagonistis Effects of Bisphenols on gmEra

In line with studies of ER/Era from other species, we showed that BPA was able to activate the Atlantic cod Era. BPA-activated gmEra with an *E*_max_ of 19-fold activation and with an EC_50_ of 1.9 μM. The potency of BPA to gmEra is comparable to potencies determined for Er from other fish species and humans, which have reported EC_50_ values in the range of 0.59 μM (in medaka^58^) to 26 μM (in carp^59^), and with human Era having an EC_50_ of 0.63 μM.^13^ However, it must be noted that direct comparison of EC_50_-values across different species and studies is challenging due to the variety of methodologies that have been used to obtain these results. Eight of the BPA analogs assessed in this study were able to transactivate gmEra as partial agonists, and four of these were found to be more potent than BPA (REP_50_-values: BPC2 > BPAF > BPTMC > BPB > BPA > BPZ > BPS). On the other hand, only BPE and BPF produced higher efficacies in comparison to BPA (*E*_max_: BPE > BPF > BPA). Our data are in accordance with previous studies on human ERa, which also demonstrated that BPA, BPB, BPC2, BPE, BPF, BPS, BPZ, and BPAF were able to activate hsERa. In addition, it was shown that BPC2, BPAF, and BPB were more potent hsERa agonists in comparison to BPA, which also aligns well with our results on gmEr.^20,32,60,61^ Experimental studies investigating the interaction of fish Er with BPA analogs are still scarce. However, it has previously been shown that BPAF is more potent than BPA in activating Era from zebrafish,^62^ and that BPF and BPB were more potent than BPA in activating medaka Era.^58^*In silico* docking analysis with three zebrafish Er subtypes identified BPAF to have a higher binding potential than BPA for binding to zfEra, zfErb1, and zfErb2, whereas BPF and BPS were found to have weaker binding potentials.^63^ These *in silico* data are also in accordance with our experimental results with gmEr.

BPA and the BPA analogs were also assessed with the luciferase reporter gene assay in combination with basal activation by EE2 (EC_20_) to investigate potential antagonistic effects on gmEra. BPC2, BPG, BPZ, and BPTMC demonstrated antiestrogenic properties by significantly decreasing the EE2-mediated activity. On the other hand, BPA, BPB, BPE, BPF, BPS, BPAF, and BADGE produced no antiestrogenic effects in this assay, although BPE and BPF appeared to produce a weak additive effect with EE2. In contrast to our findings, BPAF was previously found to have antiestrogenic effects on both hsERa and hsERb, while BPZ was shown to have no antiestrogenic properties.^20^ These data suggest that there are also species-specific differences regarding the activation potentials of orthologous ERa/Era.

### Many Bisphenols Produce Estrogenic Effects in Cod Liver Ex Vivo

The estrogenic effects of BP exposure were also investigated with Atlantic cod PCLS cultures. Most of the BPA analogs that were estrogenic in the luciferase reporter gene assay also induced Vtg production in PCLS, although the increased Vtg production after BPZ and BPFL exposure was not statistically significant. BPE and BPF, which demonstrated the highest efficacies (*E*_max_) in the gmEra luciferase reporter assay, also induced the highest Vtg-levels *ex vivo*. Induction of Vtg production by BPA exposure has previously been reported from *in vitro* studies with hepatocytes from carp and trout.^15^ Furthermore, similar results have also been obtained in *in vivo* studies with several teleosts, including Atlantic cod. In line with the *ex vivo* results reported here, juvenile Atlantic cod exposed *in vivo* to 50 μg/L waterborne BPA demonstrated elevated levels of both Vtg and the Zrp eggshell protein in plasma.^64^ BPA analogs have also been assessed in *in vivo* exposure studies with zebrafish, showing that BPAF and BPF increased Vtg synthesis.^62,63^ The results obtained from these *in vivo* experiments are in accordance with the *ex vivo* and *in vitro* data from Atlantic cod reported here.

Interestingly, BPG, which only acted as an antagonist of gmEra in the luciferase reporter gene assay, displayed estrogenic properties by increasing Vtg-levels in the PCLS. This discrepancy may point to a possible role of hepatic biotransformation enzymes in converting this compound into a metabolite that is a favorable agonist of the gmEra receptor.^65−67^ This has previously been shown for the Era antagonist tamoxifen, which in some mammalian species can be metabolized into 4-hydroxytamoxifen that further lose its alkylaminoethane side chain to form the estrogenic compounds metabolite E or tamoxifen bisphenol.^68^ However, it cannot be ruled out that BPG rather interact with and activate Erb subtypes or membrane-bound estrogen receptors (GPER) in cod liver slices, which previously have been demonstrated to be able to influence Vtg synthesis in other teleosts.^69−71^ Notably, BADGE was the only BPA analog that demonstrated antiestrogenic effects in the *ex vivo* assay by significantly reducing the amount of Vtg levels in the PCLS media. This is interesting, as BADGE did not antagonize EE2-mediated gmEra activity in the luciferase reporter gene assay. However, in previous studies, BADGE was reported to be an estrogen receptor antagonist in carp primary hepatocytes.^72^ It is therefore not unlikely that BADGE is metabolized by biotransformation enzymes into an antiestrogenic metabolite in fish liver cells.^73^ Another possibility is that the effect seen is caused by impurities in the commercial BADGE available.^74^

### Bisphenols Produce More gmAra Antagonism Than Agonism

In contrast to the agonistic effects that were observed with the majority of the BPs and gmEra, gmAra was only significantly activated by two of the BPA analogs assessed in this study, i.e., BPC2 and BPAF. Accordingly, both BPC2 and BPAF were previously identified as Ar agonists.^60^ On the other hand, both BPA and most of the BPA analogs acted as antiandrogens by significantly inhibiting the basal testosterone-mediated activity of gmAra (ranking based on IC_50_ values: BPAF > BPC2 > BPA = BPB > BPG > BPE > Flutamide > BPZ > BPF > BPTMC). In accordance with our data, BPA has previously also been shown to possess antiandrogenic effects in luciferase reporter gene assays with fathead minnow Ar.^75^ However, to our knowledge, no other studies investigating the effects of bisphenol analogs on androgen receptor activity from fish have been reported. Thus, our study represents the first extensive investigation of the agonistic and antagonistic effects of BPA analogs on the androgen receptor in a teleost species.

Antagonistic effects of several bisphenols have previously been observed using hsAR.^20,32,76^ However, the antiandrogenic potencies of the BPs seem to differ between human and cod receptors, e.g., with BPAF being the most potent gmAra antagonist, while it was the weakest antagonist of hsAR.^20^ On the other hand, BPC2, which was found to be a weak agonist and one of the most potent antiandrogenic compounds in our study, was also identified as a slightly positive hsAR agonist and a more potent antiandrogen than BPA.^60^

An apparent increase in testosterone-mediated activity of gmAra was observed when exposed to BPS and BPFL. The exact mechanism behind these observations is not yet known, but they may suggest potentiating properties of these bisphenols mediated through ligand–receptor interactions that take place elsewhere than the canonical ligand binding pocket of gmAra. We have previously observed such effects in a study with the Atlantic cod vitamin D receptor exposed to a combination of calcitriol (endogenous ligand) and certain polycyclic aromatic hydrocarbons (PAH).^77^ Similar observations of potentiating effects were also observed with a binary mixture of perfluorooctanesulfonate (PFOS) and perfluorooctanoic acid (PFOA) on the gmPpara1 receptor. In that case, PFOS alone did not activate gmPpara1, but together with PFOA, PFOS potentiated the receptor activation mediated by PFOA. Ligand-docking and molecular dynamics simulations suggested that PFOS binds to an allosteric binding site and that upon binding stabilizes an active conformation of the gmPpara1 receptor.^40^ Similar interactions may occur with gmAra and BPS/BPFL.

### Structure–Activity Relationships of Bisphenols

BPA, BPB, BPE, and BPF produced the highest efficacies as agonists for the gmEra. Common for these compounds is that they do not have extensive branching of chemical groups on the bridging carbon atom between the phenolic rings in their structures. Moreover, BPs with larger chemical groups on the bridging carbon (BPC2, BPS, BPZ, BPFL, BPTMC), as well as BPs lacking the phenolic groups in *para* positions (BADGE) or having ortho-substitutions (BPG), did not possess strong estrogenic properties in our experiments. A notable exception is BPAF, which demonstrated a slight activation of gmEra. These results are consistent with the different binding modes of agonistic and antagonistic compounds to the LBD of the human ERa, resulting in distinct conformational changes.^78^ gmEra shares 58% sequence identity with hsERa in the LBD, and all the residues shown to be involved in the binding of estradiol are positionally conserved, suggesting similar ligand-binding properties (Figure S4). Furthermore, structural and functional studies showed that unhindered phenolic groups in the *para* position and favorable hydrophobic substituents are necessary for the efficient binding and estrogenic activity of an agonist.^78^ Similarly, for BPA analogs, the *p*-hydroxyl group on unhindered phenolic groups and a hydrophobic group on the bridging carbon favor estrogenic activity.^13,72^ Crystal structures of bisphenols bound to hsER have shown that, depending on the substituents on the bridging carbon, bisphenols can assume agonist or antagonist binding modes.^79^ Thus, the low efficacy of BPAF and the lack of estrogenic effects of BPFL (which both have large substituent groups on the bridging carbon) and the antiestrogenic effects of BPG (which has ortho-substitutions on the phenolic groups) could be explained by these unfavorable structural features. The antagonistic effects of BADGE could be due to the large substituents on the phenolic hydroxy groups (see structures in Figure 1). BADGE was also found to inhibit E2-induced Vtg synthesis in carp hepatocytes, and it was suggested to be an antagonist.^72^ Among the BPA analogs that showed antiestrogenic effects in the reporter assays, BPC2, BPZ, and BPTMC possess large substituents on the bridging carbon atoms, which likely result in antagonist-like conformational changes, similar to the bisphenol analog BPC.^79^

None of the bisphenols assessed in this study demonstrated strong androgenic effects. However, both BPC2 and BPAF were able to significantly activate the gmAra receptor, in contrast to what was observed with the gmEra receptor. On the other hand, we observed antiandrogenic effects by all the bisphenols, except BPS, BPFL, and BADGE. Based on *in silico* modeling, it has been suggested that bisphenols bind to human AR in antagonist-like orientations,^79^ which also agrees with the antiandrogenic effects of most of the bisphenols observed here and in earlier reports, as well as with the strong sequence identity (62%) shared between hsAR and gmAra in the LBD (Figure S5).^13^ Functional assays and molecular modeling of binding of bisphenol analogs to hsAR also suggested that the phenolic hydroxyl groups and hydrophobic interactions by hydrophobic substituents on bridging carbon atom favor antiandrogenic activity.^13,80^ The antiandrogenic activity of bisphenols can also be affected by substituents on the phenolic ring and the bridging carbon.^13^

BPA and the majority of the BPA analogs assessed in this study exhibited endocrine-disrupting properties *in vitro* and/or *ex vivo* by either activating or inhibiting the gmEr and gmAr receptors. Moreover, BPB, BPE, and BPF displayed higher or similar efficacies as BPA in activating the gmEr, while some of the BPs (BPB, BPC2, and BPAF) were more potent agonists for gmEr than BPA. Notably, BPG and BADGE demonstrated discrepancies in their estrogenic properties between the *in vitro* and *ex vivo* assays. Neither BPG nor BADGE influenced the gmEr activity in the luciferase reporter gene assay. However, in the *ex vivo* exposures with PCLS, BPG, and BADGE produced estrogenic and antiestrogenic effects, respectively. This suggests a role for hepatic biotransformation in producing metabolites with altered chemical structures that favor interactions with gmEr. While only BPC2 and BPAF were able to activate the gmAr, a majority of the BPs demonstrated antiandrogenic effects by inhibiting gmAr activity. Importantly, this study represents the first comprehensive analysis of BPA analogs on the androgen receptor in any fish species, emphasizing that the androgen receptor may be an important molecular target of BPs and that modulation of Ar activity can be a common mode of action caused by BP exposures. Our work with Atlantic cod Era and Ara provides supporting evidence of the endocrine-disruptive effects that aquatic organisms may experience from BP exposures. Endocrine and reproductive effects can manifest over time at higher biological levels, potentially influencing population structures. Additionally, it is important to acknowledge that BPs replacing BPA, including the most common BPA replacement compounds such as BPF, BPS, and BPAF, exhibit similar or greater endocrine disruptive properties compared with BPA. Therefore, if used without stringent regulations, these bisphenols pose a persistent risk to both environmental and human health.

## Supporting Information Available

(Table S1) Primers used for cloning of Atlantic cod estrogen receptor (esr1) and androgen receptor (ara); (Figures S1–S5) Western blot analyses of gmEra and gmAra in transiently transfected COS-7 cells, cytotoxicity analyses of COS-7 cells exposed to bisphenols, viability analyses of Atlantic cod precision-cut liver slices exposed to bisphenols, sequence alignments of the ligand binding domain of gmEra and gmAra, and their human orthologs (PDF)
