# Supplementary material for: Interaction of Bisphenol A and Its Analogs with Estrogen and Androgen Receptor from Atlantic Cod (Gadus morhua)
Source: Environ Sci Technol. 2024 Aug 1;58(32):14098–109. doi: 10.1021/acs.est.4c01500 (PMC11325555; doi:10.1021/acs.est.4c01500)
Supplement: Supplementary file 1 — es4c01500_si_001.pdf [file es4c01500_si_001.pdf]

Supporting information

Interaction of bisphenol A and its analogs with estrogen and androgen receptor from Atlantic cod (*Gadus morhua*)

Siri Øfsthus Goksøyr[1], Fekadu Yadetie[1], Christine Tveiten Johansen[1], Rhîan Gaenor Jacobsen[1], Roger Lille-Langøy[1], Anders Goksøyr[1], Odd André Karlsen[1,]*

[1]Department of Biological Sciences, University of Bergen, N-5020 Bergen, Norway

**Corresponding author, e-mail: odd.karlsen@uib.no*

Summary: 5 Pages, 1 Table, 5 Figures

Supplementary Table S1. Primers used for cloning of estrogen receptor 1 (esr1) and androgen receptor (ara) cDNA

Primer name	Primer sequence (5´-3´)
Cod Esr1 hinge EcoRI fwd	gaattcATGAAAGGAGGTATGCGCAAGG
Cod Esr1 BamHI rev	ggatccTCAGACGGACGCACACTCGTG
Cod Ar hinge EcoRI fwd	CCGgaattcGGCATGAGCCTCAAAGGTCG
Cod Ar hinge BamHI rev	GGCggatccCTAGCCTGCCTTATGGAAAAG

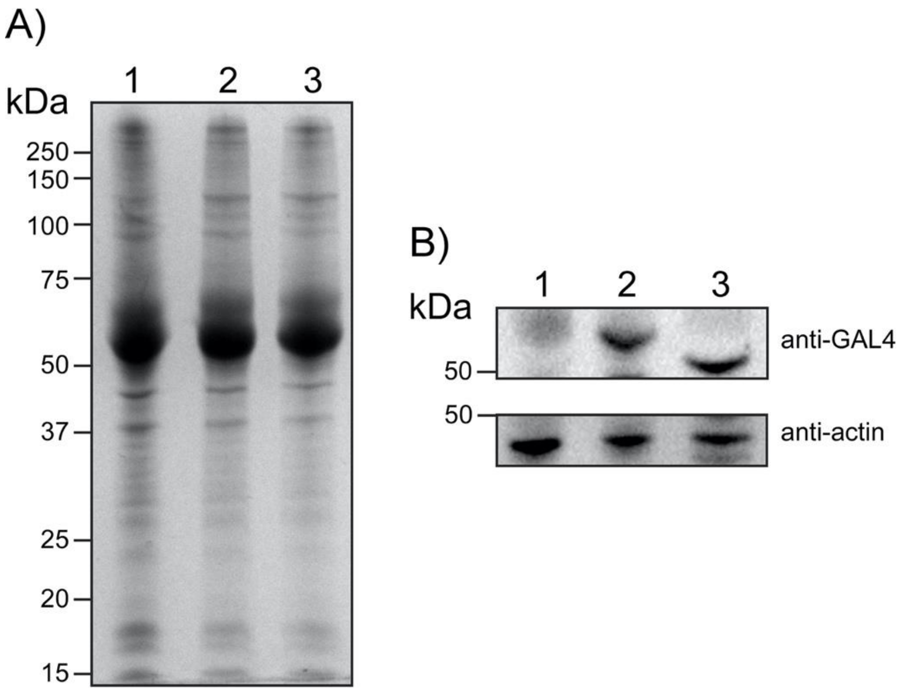

Figure S1. Synthesis of gmEra and gmAra in COS-7 cells. Protein synthesis of gmERa and gmAra in COS-7 cells transfected with pCMX-GAL4(DBD)-Era(LBD) and pCMX-GAL4(DBD)-Ara(LBD), respectively, were confirmed with SDS-PAGE and western blotting. **A)** Coomassie-stained polyacrylamide gel of untransfected COS-7 cells **(lane 1)**, COS-7 cells transfected with gmEra **(lane 2)** and COS-7 cells transfected with gmAra **(lane 3)**. **B)** Upper panel: western blot corresponding to the gel shown in **A)** using anti-GAL4 antibody for detection of the GAL4-gmEra (**lane 2)** and GAL4-gmAra (**lane 3)** fusion proteins. Untransfected COS7 (**lane 1)** cells were

used as a negative control. Lower panel: Mouse-anti-beta actin antibodies were used for monitoring protein loading.

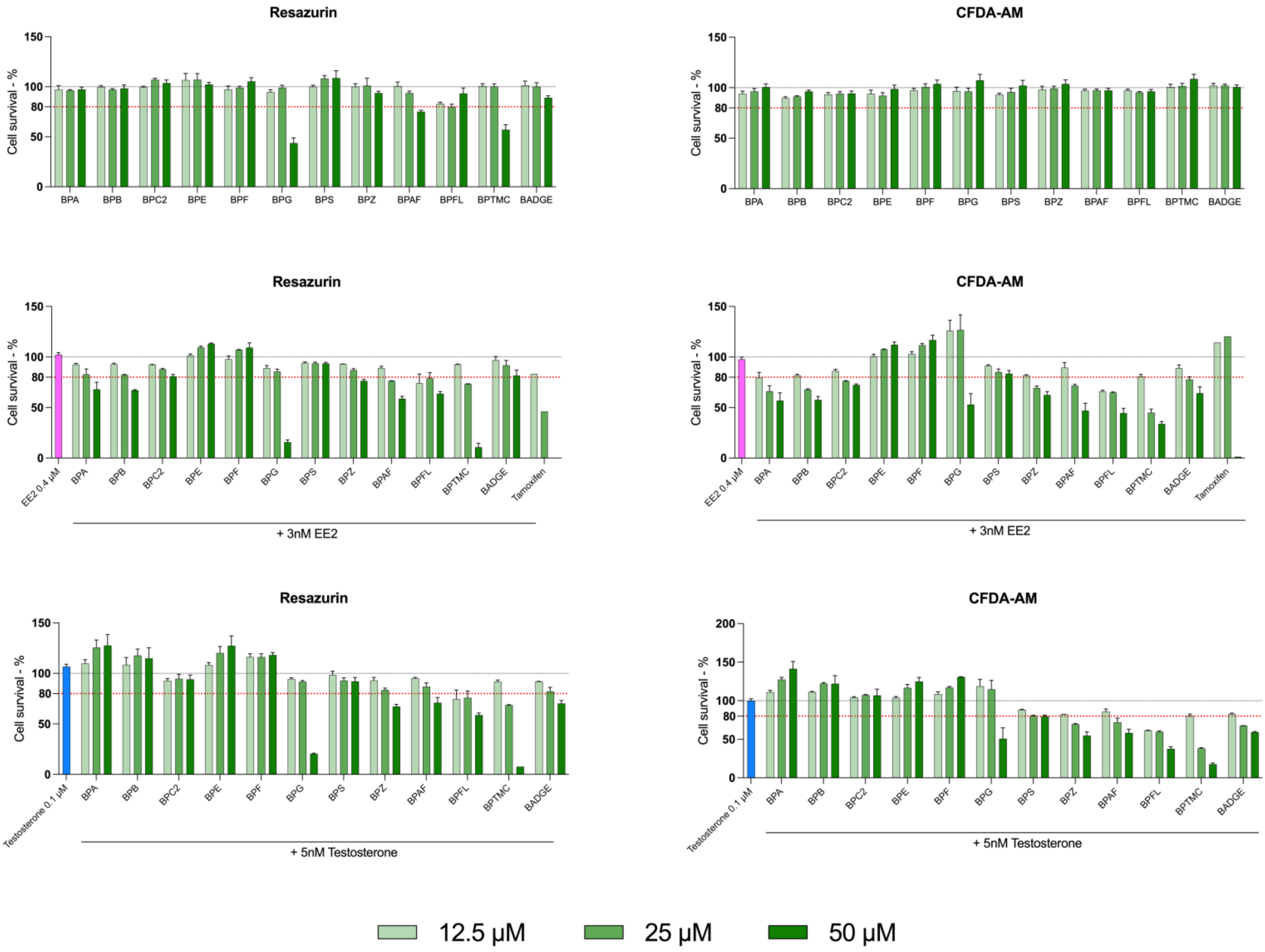

Figure S2. Assessing metabolic activity (resazurin) and cell membrane integrity (CFDA-AM) in COS-7 cells after BP exposures. COS-7 cells were exposed to three concentrations of each BP, as well as three concentrations of the BP mixtures with testosterone or EE2, as used in the luciferase reporter gene assays. COS-7 cells were exposed at 37 °C and 5 % CO_2 for 24 hours. Cytotoxicity is indicated by a reduction in the fluorescent signal (denoted as % Cell survival) relative to the solvent control (DMSO, dotted line) adjusted to 100 %. A reduction in cell viability exceeding 20 % is generally regarded as cytotoxic (indicated with a red dotted line) (ISO, I. 2009. 10993–5: 2009 Biological evaluation of medical devices—part 5: tests for in vitro cytotoxicity. International Organization for Standardization, Geneva.).

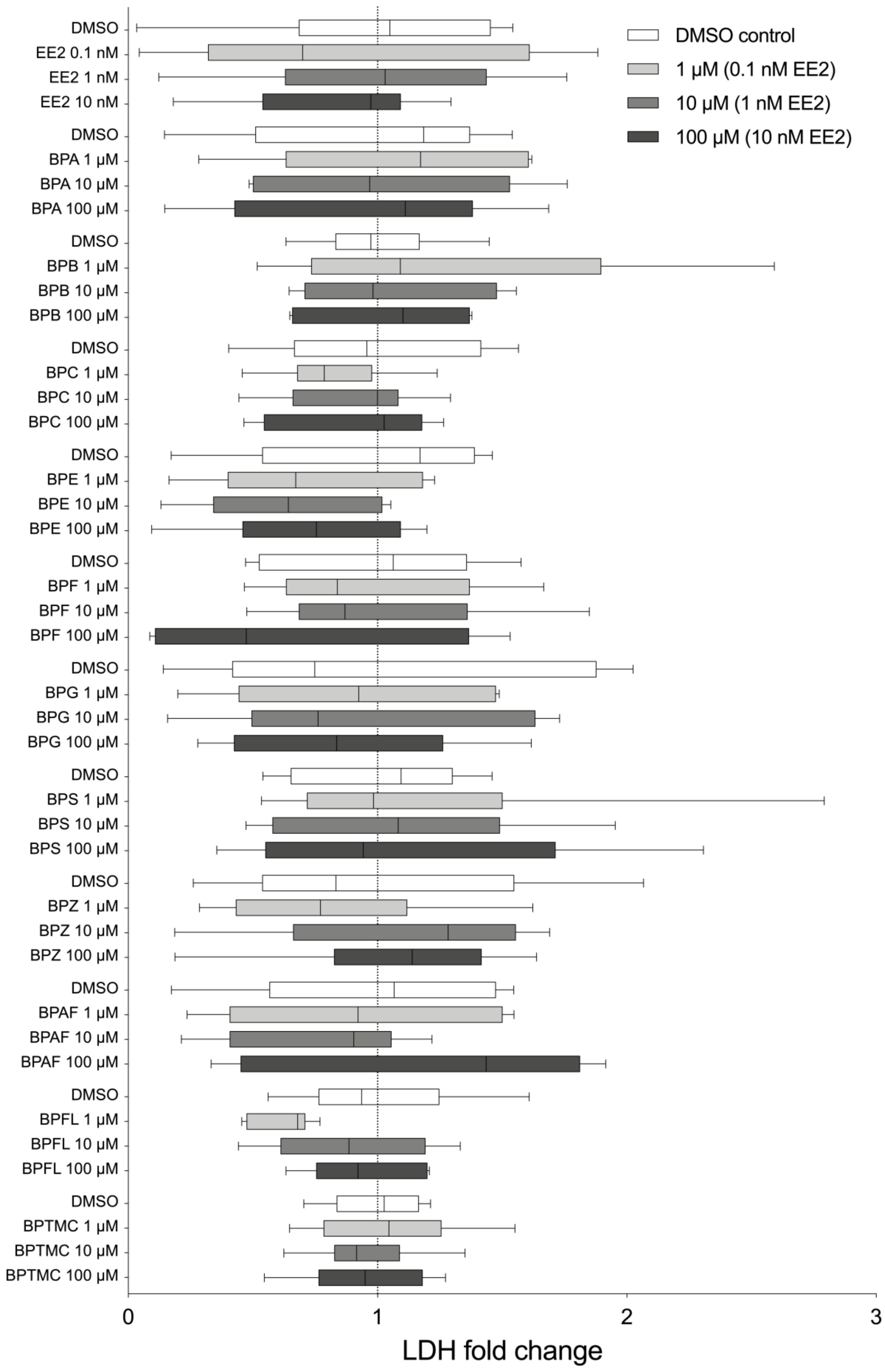
DMSO control
1 µM (0.1 nM EE2)
10 µM (1 nM EE2)
100 µM (10 nM EE2)
DMSO
EE2 0.1 nM
EE2 1 nM
EE2 10 nM
DMSO
BPA 1 µM
BPA 10 µM
BPA 100 µM
DMSO
BPB 1 µM
BPB 10 µM
BPB 100 µM
DMSO
BPC 1 µM
BPC 10 µM
BPC 100 µM
DMSO
BPE 1 µM
BPE 10 µM
BPE 100 µM
DMSO
BPF 1 µM
BPF 10 µM
BPF 100 µM
DMSO
BPG 1 µM
BPG 10 µM
BPG 100 µM
DMSO
BPS 1 µM
BPS 10 µM
BPS 100 µM
DMSO
BPZ 1 µM
BPZ 10 µM
BPZ 100 µM
DMSO
BPAF 1 µM
BPAF 10 µM
BPAF 100 µM
DMSO
BPFL 1 µM
BPFL 10 µM
BPFL 100 µM
DMSO
BPTMC 1 µM
BPTMC 10 µM
BPTMC 100 µM
0
1
2
3
LDH fold change

Figure S3. Assessment of PCLS tissue viability in BP-treated and DMSO control groups. Data points represent mean ± SD of fold-changes (normalized to DMSO) in LDH activity (in the culture medium) per mg of tissue (n = 5-6). For each compound, increasing concentrations used are arranged bottom up vertically (y-axis), starting with DMSO control. None of the treatment groups had statistically significantly increases in LDH activity in comparisons between the different concentrations and the corresponding DMSO control. One treatment group (BPFL, 1µM) had significantly lower LDH activity ($p < 0.05$).

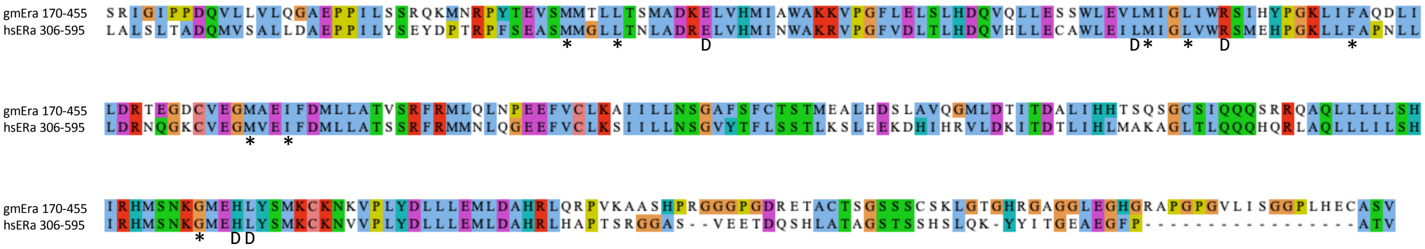

Figure S4. Sequence alignment of ligand binding domain of gmEra and hsERa. Alignment was constructed in Jalview using Clustal with default settings. Amino acid residues involved in direct binding of estradiol is indicated with (Δ), while (*) indicates amino acids that are part of the ligand binding pocket. The alignment is coloured using Clustal colour scheme. ("Clustal W and Clustal X version 2.0" Bioinformatics, 23: 2947 (2007)).

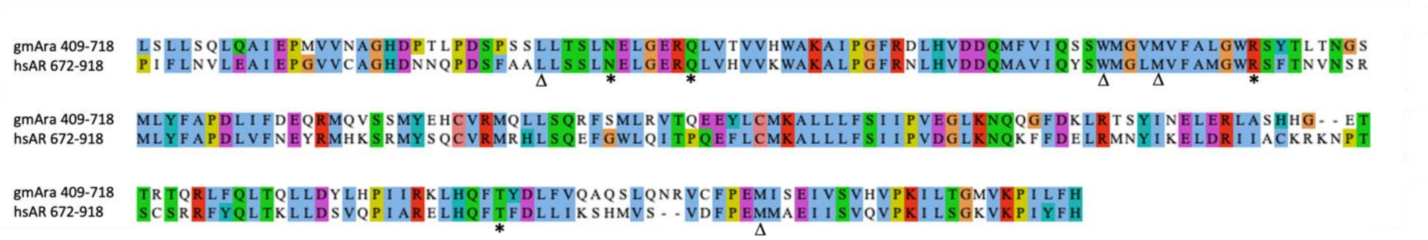

Figure S5. Sequence alignment of ligand binding domain of hsARa and gmAra. Alignment was constructed in Jalview using Clustal with default settings. Amino acid residues involved in direct binding of estradiol is indicated with (Δ), while (*) indicates amino acids that are part of the ligand binding pocket. The alignment is coloured using Clustal colour scheme. ("Clustal W and Clustal X version 2.0" Bioinformatics, 23: 2947 (2007)).
